# Polystyrene nanobeads exacerbate chronic colitis in mice involving in oxidative stress and hepatic lipid metabolism

**DOI:** 10.1186/s12989-023-00560-8

**Published:** 2023-12-18

**Authors:** Juan Ma, Yin Wan, Lingmin Song, Luchen Wang, Huimei Wang, Yingzhi Li, Danfei Huang

**Affiliations:** State Key Laboratory of Food Science and Resources, International Institute of Food Innovation, China-Canada Joint Lab of Food Science and Technology (Nanchang), Key Laboratory of Bioactive Polysaccharides of Jiangxi Province, Nanchang University, 235 Nanjing East Road, Nanchang, 330047 China

**Keywords:** Polystyrene nanobeads, Chronic colitis, Oxidative stress, Liver metabolism, Lipid metabolism

## Abstract

**Background:**

Nanoplastics (NPs) are omnipresent in our lives as a new type of pollution with a tiny size. It can enter organisms from the environment, accumulate in the body, and be passed down the food chain. Inflammatory bowel disease (IBD) is a nonspecific intestinal inflammatory disease that is recurrent and prevalent in the population. Given that the intestinal features of colitis may affect the behavior and toxicity of NPs, it is imperative to clarify the risk and toxicity mechanisms of NPs in colitis models.

**Methods and results:**

In this study, mice were subjected to three cycles of 5-day dextran sulfate sodium (DSS) exposures, with a break of 7 to 11 days between each cycle. After the first cycle of DSS exposure, the mice were fed gavagely with water containing 100 nm polystyrene nanobeads (PS-NPs, at concentrations of 1 mg/kg·BW, 5 mg/kg·BW and 25 mg/kg·BW, respectively) for 28 consecutive days. The results demonstrated that cyclic administration of DSS induced chronic inflammation in mice, while the standard drug “5-aminosalicylic acid (5-ASA)” treatment partially improved colitis manifestations. PS-NPs exacerbated intestinal inflammation in mice with chronic colitis by activating the MAPK signaling pathway. Furthermore, PS-NPs aggravated inflammation, oxidative stress, as well as hepatic lipid metabolism disturbance in the liver of mice with chronic colitis.

**Conclusion:**

PS-NPs exacerbate intestinal inflammation and injury in mice with chronic colitis. This finding highlights chronically ill populations’ susceptibility to environmental hazards, which urgent more research and risk assessment studies.

**Supplementary Information:**

The online version contains supplementary material available at 10.1186/s12989-023-00560-8.

## Background

With the widespread use of plastic products, plastic waste entering the environment is steadily increasing [1]. According to ISO/TR 21960:2000, plastic particles with diameters of 1-1000 μm are classified as microplastics (MPs), while those below 1 μm as nanoplastics (NPs) [2]. MPs/NPs can be found in the environment as either primary or secondary MPs/NPs. Primary MPs/NPs are initial plastic particles present before they enter the environment, with origins ranging from toothpaste and cosmetics [3] to air blast technology and carriers for drugs in medicine. Secondary MPs/NPs are fragmented particles generated from larger-sized plastic products due to physical, chemical and biological processes in the environment [4]. Consequently, actual MPs/NPs in the environment were highly heterogeneous, containing a variety of morphologies such as fiber, fragment, foam, film, and bead as well as a wide range of materials such as low/high-density polyethylene (PE), polypropylene (PP), polyethylene terephthalate (PET), polystyrene (PS), polyvinyl chloride (PVC), etc. [5]. Because of their small size and non-degradable nature, MPs/NPs provide a long-term hazard to ecosystems and can be consumed unwittingly or inadvertently by a wide range of species, causing physical and physiological risks [6]. Studies have shown that MPs made zebrafish hyperactive, leading to a reduction in glucose and acetaldehyde metabolites [7]. Exposure of macroinvertebrate *Corbicula fluminea* to MPs resulted in behavioral toxicity and chronic bio toxicity [8]. Data from several studies suggested that MPs can disperse and accumulate in mouse tissues, resulting in aberrant energy and lipid metabolism, oxidative stress [9], lowered maternal mice and altered reproduction and development in offspring mice [10]. Furthermore, MPs/NPs can act as transporters for other pollutants in the environment, inflicting much greater damage. According to Lu et al., MPs can enhance cadmium toxicity in zebrafish, and cadmium and MPs co-exposure can cause oxidative damage and inflammation [11].

According to previous studies, MPs/NPs can enter the circulatory system and tissues via the intestinal epithelium, triggering inflammatory responses and necrosis [12, 13]. Several 5–10 μm MPs fragments with spherical or irregular shapes have been found in the human placenta and thrombi [14]. Similarly, laboratory studies also found that 5 μm MPs, rather than 20 μm, can transfer into visceral tissue showing that smaller particles are likely to penetrate and remain in tissue. Because of NPs’ drastically enhanced properties, reactivity and effect [15], additional concerns regarding NPs and their environmental and human health dangers have risen. As there are no effective methods for the determination, quantification and assessment of NPs in real environments, commercially available model particles were used to determin the size-specific toxicity.

As a chronic inflammatory disorder of the gastrointestinal tract, inflammatory bowel disease (IBD), including ulcerative colitis (UC) and Crohn’s disease, is becoming increasingly prevalent globally and disturbs the quality of life of those who suffer from it [16]. It is distinguished by diffuse inflammation of the colorectal mucosa and damage to the epithelial layer [17]. Environmental factors, such as food additives as well as pollutants are increasingly being linked to the aggravation of IBD, most likely due to increased amounts of contaminants entering the body through the intestinal barrier, causing deterioration of other physiological sub-systems in the body [18]. Therefore, more research on environmental factors in IBD and how they interact is needed.

Given that the intestinal features of colitis may affect the behavior and toxicity of MPs, it is imperative to clarify the risk and toxicity mechanisms of NPs in gastrointestinal dysfunction models. Compared with acute colitis, circulating drug delivery can mimic the recurrent morbidity of chronic colitis, which more closely fits the pathogenesis and mechanism of human IBD and makes our study more convincing. Here, mice were given cyclic DSS intragastrically and simultaneously treated with various amounts of polystyrene nanobeads (PS-NPs), biochemical analyses of colon tissue and metabolomics/lipidomics analyses of the livers were then employed. The main objective of this study was to reveal the effect of PS-NPs exposure on mice with chronic colitis. This study gives a warning for NPs in the chronic colitis population, as well as a reminder to people to pay more attention to the potential harm caused by environmental pollutants to the chronic disease population.

## Materials and methods

### Material characterization

Pristine polystyrene nanobeads (PS-NPs) selected for this experiment were purchased from Oisheng Plastic Raw Materials Co., LTD. Dongguan City, Guangdong Province, China. They are uniform polystyrene particles with a diameter of 0.1 μm, which were characterized in our previous study [19]. Dextran Sodium Sulfate (DSS) for mouse modeling, purchased from MP Biomedicals (MP, USA). 5-Aminosalicylic acid (5-ASA) was used as a reagent for the standard drug group and purchased from Shanghai Maclean Biochemical Technology Co. Ultra-pure water was used for the preparation of all reagents in the experiment.

### Experimental exposure

**Fig. 1:**
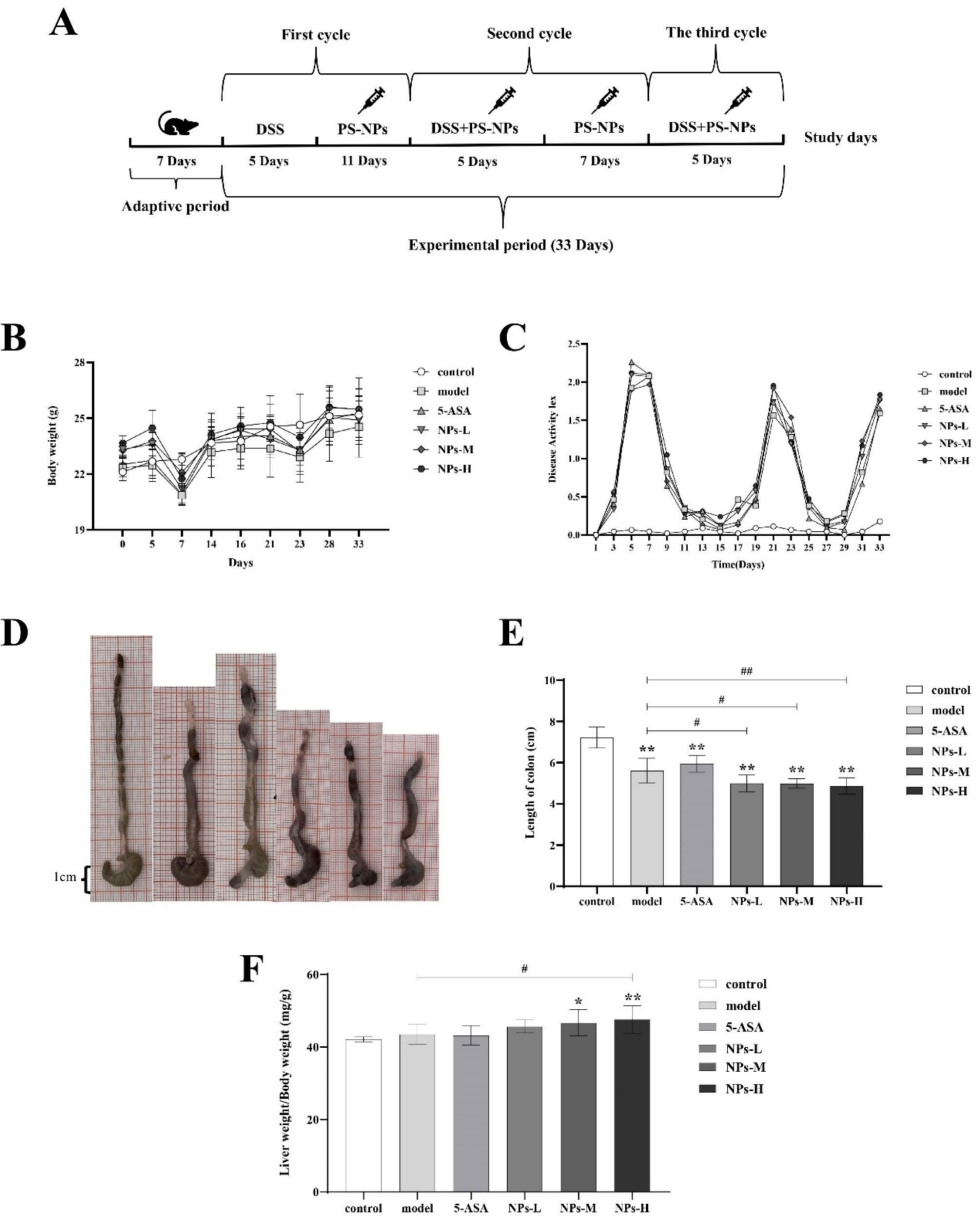
The growth phenotype of mice. (**A**) experimental design of the toxicity of PS-NPs on mice with chronic colitis. (**B**) Body weight. (**C**) Disease activity index score sheet. (**D**) Gross morphological changes in the colon. (**E**) Colon length. (**F**) Liver coefficient. Data are presented as mean ± SD, n ≥ 9. One-way ANOVA and Tukey’s multiple comparison test. The asterisk and pound represents statistically significant difference (*p < 0.05, **p < 0.01 compared to the control group; ^#^*p* < 0.05, ^##^*p* < 0.01compared to the model group)

A mouse model of chronic colitis was established by subjecting mice to three cycles of 5-day DSS exposure, with 7 to 11 days between each cycle. The experimental design is shown in Fig. 1A. Briefly, ninety 7-week-old male C57BL/6J mice were purchased from Hunan Sia Laboratory Animal Co., LTD (Hunan, China) and placed in an environment- controlled facility (temperature 22 ± 2 °C, humidity 55 ± 10%), with a photoperiod of 12 h light / 12 h dark cycle. They could obtain food and water ad libitum. After a week of acclimatization feeding, 75 mice were given oral cyclic administration of DSS in drinking water to induce chronic colitis, while the remaining 15 mice were left untreated and received daily intragastric water infusions from Day 6 (the control group). 75 mice received 2.5% DSS in drinking water for 5 days and then purified water for 11 days in Cycle One, followed by lower doses of DSS in drinking water (2%) for 5 days and purified water for 7 days in Cycle Two, and finally 2% DSS in drinking water for 5 days [20]. After the first cycle of DSS exposure, 75 mice to induce chronic colitis were randomly divided into five groups of 15 mice each, and daily received intragastric infusions of water (the model group), 5-aminosalicylic acid (the 5-ASA group, 200 mg/kg·BW) [21], or three different concentrations of PS-NPs (the NPs-L group, 1 mg/kg·BW; the NPs-M group, 5 mg/kg·BW and the NPs-H group, 25 mg/kg·BW) for 28 days. The dose of PS-NPs was determined based on previous studies [22, 23]. The dose was calculated based on the body weight (20 g) of the mice. The water consumption and body weight of the mice were recorded daily and their condition was observed. The fecal consistency and and occult blood level of mice were recorded.

After 28 days of exposure, the blood of mice from all the groups was collected using the eyeball extraction method for serum biochemical analysis. The livers and colons were quickly weighed after removal and then frozen in dry ice or non-frozen tissue RNA preservation solution (Beijing Solarbio Technology Co., China) for subsequent analysis. The experiments were approved by the animal experiment ethics committee of Nanchang University.

### Histopathological analysis of liver and colon tissues

Murine liver and about 5 mm of the distal end of colon tissues were fixed with a 4% paraformaldehyde solution followed by dehydration with varied alcohol concentrations. Tissue blocks were made transparent by substituting xylene for alcohol and placed in melted paraffin wax, which was completely absorbed by the tissue before being cooled and solidified into blocks. The embedded wax blocks were cut into thin slices with a slicer, then blanched and dried. The dried paraffin slices were dewaxed with xylene, hydrated with ethanol and stained with hematoxylin and eosin (H&E). Finally, images were captured and analyzed using a RoW-real-time Digital Pathology System (Aperio LV1, Leica Microsystems Ltd., Germany).

### Biochemical tests of the serum

Blood samples were coagulated naturally for 2–3 h at room temperature followed by centrifugation at 3000 rpm for 10 min. The supernatant was collected and stored at -80 °C before use. 80 μL of serum was taken and mixed with 160 μL of normal saline. The contents of alanine aminotransferase (ALT), cholesterol (CHOL) and glucose (GLUC) in serum were analyzed by an AU480 Chemistry Analyzer (Beckman Coulter, USA).

### Evaluation of oxidative stress in liver tissue

The homogenate was prepared by mixing tissue (mg) with normal saline or Western and IP cell lysates (μL) at a ratio of 1:10. Then the homogenate was frozen and centrifuged according to the requirements of the kit. The supernatant was taken for determination. The contents of malondialdehyde (MDA), superoxide dismutase (SOD), total antioxidant capacity (T-AOC) and reduced glutathione (GSH) in the liver were determined by using commercial kits (China Biyuntian Biotechnology Co., LTD.). A BCA protein concentration assay kit was used for protein quantification.

### Q-PCR analysis of colon tissue

Weighed 50 mg of colonic tissue in a 2 mL RNase-free tube. Added 1 mL of Trizol reagent to each sample, ground it on ice, and then kept it in the ice bath for 5 min. After adding 200 μL of chloroform, the homogenate was shaken vigorously for 15 s, kept in the ice bath for 3 min, and then centrifuged for 15 min at 4 °C at 12,000 g. Transferred 400 μL of supernatant to a new 2 mL RNase-free tube, added 400 μL of isopropanol, mixed it upside down, and then kept it on ice for 10 min, and then centrifuged for 15 min at 4 °C at 12,000 g. After removal of the supernatant, 1 mL of 75% ethanol was added and mixed, then centrifuged for 15 min at 4 °C at 7500 g. Discarded the supernatant, let the RNA precipitate dry at room temperature for 10 min until it resembled slightly moist and gel-like, then re-dissolved in 50 μL of sterile enzyme-free water at 50 °C ~ 60 °C and blown evenly.

The quality of extracted RNA was evaluated by using NanoDrop™ 2000 spectrophotometer (Thermo Scientific, USA) at 260/280 nm absorbance. To verify RNA integrity, extracts were fractionated by electrophoresis using 1.2% agarose gel (containing ethidium bromide). The qualified RNA was diluted to uniform concentration, and the RNA was reverse transcribed into cDNA according to the reverse transcription kit method, and then q-PCR experiments were performed according to the q-PCR kit. The primer sequences are given in Table 1.

**Table 1:** Primer sequences used for real-time qPCR

Gene	Direction	Primer sequence (5’-3’)	Tm(°C)
β-actin TNF-α IL-10	Forward Reverse Forward Reverse Forward Reverse	ACTGCCGCATCCTCTTCCTC AAAGAGCCTCAGGGCATCGG TAGCCCACGTCGTAGCAAAC GCAGCCTTGTCCCTTGAAGA AAGGGTTACTTGGGTTGCCA CCTGGGGCATCACTTCTACC	59.5 59.5 59.5 59.5 59.5 59.5

### Expression of MAPK pathway-related proteins in colon tissue

Western blot was used to detect the expression of proteins related to the mitogen-activated protein kinase (MAPK) pathway in colon tissues. Weighed an equal amount of colonic tissue of one mouse from each group. After adding protein inhibitors (100:1) to the Western and IP cell lysates, the tissues and lysates were mixed in a ratio of 1:10 (mg:μL) to homogenates. The homogenizations were ultrasonically crushed for 30 s, centrifugated for 10 min at 12,000 g, and supernatants were then taken. After adding to 5×SDS loading buffer, the supernatants were diluted to 1×, and then Metal Bath at 95 °C for 5 min.

According to the molecular weight of the protein, the separation gel with 10% concentration and the concentrated gel with 5% concentration were prepared, and the appropriate sample loading volume was selected according to the internal reference protein GAPDH. PowerPac Universal power supply (Bio-rad, USA) was selected for electrophoresis, and then Bio-rad rapid membrane transfer apparatus (Bio-rad, USA) and PVDF membrane (Millipore, USA) were selected for rapid membrane transfer. A sealed solution containing 5% BSA (Bovine serum albumin) was applied to the protein membrane, and it was agitated on a decolorizing shaker for 1 h. After sealing, the sealing solution was sucked out and the protein membrane was incubated with the primary antibody (1:1000) overnight. (The primary antibody has p44/42 MAPK (Erk1/2), Phospho-p44/42 MAPK (Erk1/2), SAPK/JNK Antibody, Phospho-SAPK/JNK, p38 MAPK (D13E1) XP and Phospho-p38 MAPK. Purchased from Cell Signaling Tachnology, China). The next day, the protein membrane was washed with TBST on a decolorization shaker a total of six times for five minutes each. After incubation with the corresponding secondary antibody for 1 h, the protein membrane was washed with TBST and then 6 times on a decolorizing shaker for 5 min each. After treating it under the instructions for the BeyoECL plus kit (Beyotime Biotechnology Co., Shanghai China) the protein membrane was then placed into a ChemiDoc Imaging system (Bio-rad, USA) to photograph it. Image Lab software was used to measure and calculate the optical density analysis of protein bands. Three parallel experiments were performed independently for each protein.

### Metabolomics and lipidomics analysis of liver tissues

50 mg liver tissue of mouse from each group was mixed with 1000 μL Watsons water and ground with a high-speed tissue grinder, followed by ultrasonic treatment for 10 min. 750 μL methanol (MeOH) and 2500 μL methyl tert-butyl ether (MTBE) were added and then vortexing for 1 min. The suspension was centrifuged (5000 rpm, 4 °C) for 15 min, and the supernatant (about 1.5 mL) was collected and dried in a rotary vacuum concentration evaporator for 1.5 h. After being re-suspended in a 50:50 (v/v) solution of MeOH and dichloromethane containing 5 mM ammonium acetate, samples were further filtered through an organic filter. About 700 μL of the filtered solution was collected, stored overnight (12 h) at -20 °C to promote protein precipitation, centrifuged (12,000 rpm, 4 °C) for 15 min to collect the supernatant, and then passed through an organic filtration membrane. Following that, the supernatant was collected in an injection vial for the purpose of analyzing water-soluble metabolite.

Liquid chromatography (LC) separation was used to perform a lipidomic study utilizing a Shimadzu Nexera X2 UHPLC system (Kyoto, Japan). The separation was performed using a Waters HSS T3 C18 column (2.1 mm×100 mm, 2 μm) at 40 °C. For MS/MS data acquisition, the MS system operates using the AB SCIEX Triple TOF 5600 quality analyzer, which has positive and negative ion modes of Information Correlation Acquisition (IDA). For metabolic analysis of the liver, LC was isolated using the Shimadzu Nexera X2 UHPLC system (Kyoto, Japan). MS/MS data were obtained using the same method as lipidomics analysis.

### Statistical analysis

Generate statistical charts with GraphPad Prism 8 (GraphPad Software Company, USA) was used for statistical analysis and visualization. All data are given as the means ± standard deviation (SD) and analyzed by one-way ANOVA combined with Tukey’s multiple comparison test. *p* < 0.05 was significant and statistically significant. *p <* 0.01 was extremely statistically significant.

## Results

### PS-NPs treatment aggravates the symptoms of DSS-induced chronic colitis in mice

The disease activity index (DAI) was calculated based on weight loss, fecal consistency and occult blood. As shown in Fig. 1B and C, DSS treatment caused significant weight loss in mice, especially on the second day after the end of the DSS feeding cycle, which was also confirmed by the DAI results. Cyclic DSS treatment significantly decreased the colon length of mice, and a more severe short was observed in mice of the PS-NPs groups (Fig. 1D, E). Additionally, the liver coefficient of mice was elevated by DSS and PS-NPs treatment (Fig. 1F). These results indicated that the chronic colitis was successfully established and partially exacerbated by PS-NPs.

### PS-NPs treatment exacerbates liver and colon histological injury in mice with DSS-induced chronic colitis

**Fig. 2:**
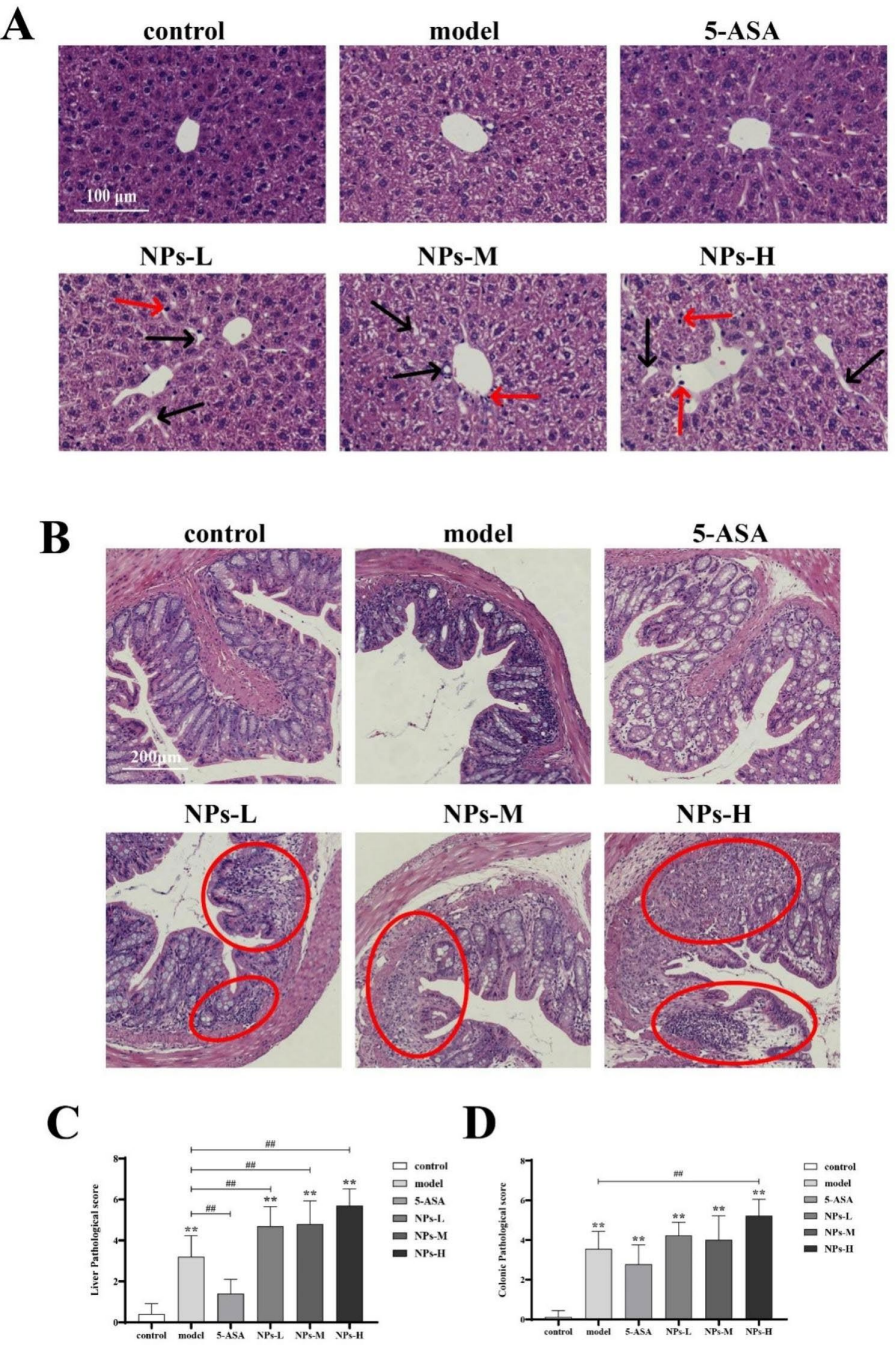
Histopathological analysis of the liver and colon. (**A**) H&E stained sections of liver (The red shear head in the figure refers to the inflammatory cell infiltrate and the black shear head refers to the fat vacuole). (**B**) H&E stained sections of colon (The red circles in the picture are severely damaged areas). (**C**) Histogram of liver tissue score. (**D**) Histogram of colon tissue score. Data are presented as mean ± SD, n ≥ 9. One-way ANOVA and Tukey’s multiple comparison test. The asterisk and pound represents statistically significant difference (*p < 0.05, **p < 0.01 compared to the control group; ^#^*p* < 0.05, ^##^*p* < 0.01 compared to the model group)

The representative images of H&E staining sections of the liver and the colon of mice from different treatments were shown in Fig. 2, respectively. The structure of the liver in normal mice was normal and showed no significant histological change (Fig. 2A). The structure of the mice liver from the model group was fuzzy, with the hepatic sinuses of hepatocytes slightly edema and dilated, accompanied by cellular inflammatory infiltration. The structure of the liver in mice of the 5-ASA group was clear and alleviated to some extent when compared with that of the model group. The histopathological characteristics of the mice liver of the low-dose NPs (NPs-L) group were similar to that of the model group, while turbidity and swelling of liver cells, increased fat vacuoles and inflammatory cell infiltration was observed in the liver of mice in the medium-dose group (NPs-M) and high-dose group (NPs-H).

As to the structure of the colon tissue, intact and normal shape was observed in normal mice, while damaged and atrophic crypts, with a small amount of inflammatory cell infiltration, were found in the mice from the model group (Fig. 2B). The 5-ASA was found to slightly alleviated destruction of the colon tissue when compared with the model group. The aggravated degree of crypt destruction, gland atrophy and inflammatory cell infiltration in colon tissue increased with increasing concentration of NPs. The liver histology (Fig. 2C) and colon histology (Fig. 2D) were scored according to the scoring criteria shown in Table 2.

**Table 2:** Scoring criteria of histological analysis

Liver histology rating indicators					
Fat vacuoles	Inflammatory cell infiltration		Hepatocytes are cloudy swelling		Score
Normal Small vacuoles Vacuoles in the cytoplasm Large vacuoles	Normal A small acount Major Vast		Normal Mild Moderate Severe		0 1 2 3
**Colon histology rating indicators**					
**Inflammatory infiltration**		**Crypt injury rate** **(CIR)**		**Score**	
Normal infiltrate the base of crypt infiltrate into the muscularis mucosa Extensive infiltration of the muscular layer infiltrate to the submucosa		0 0 < CIR ≤ 1/4 1/4 < CIR ≤ 1/2 1/2 < CIR ≤ 3/4 3/4 < CIR ≤ 1		0 1 2 3 4	

### PS-NPs treatment aggravates serum markers of liver function in mice with DSS-induced chronic colitis

**Fig. 3:**
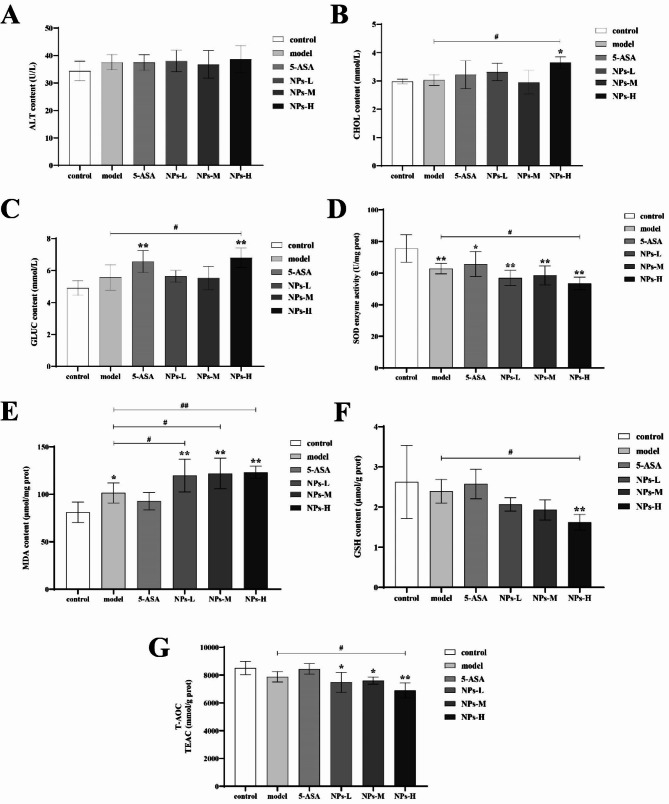
Serum biochemical analysis and liver oxidative stress parameters. (**A**) ALT. (**B**) CHOL. (**C**) GLUC. (**D**) SOD. (**E**) MDA. (**F**) GSH. (**G**) T-AOC. Data are presented as mean ± SD, n ≥ 6. One-way ANOVA and Tukey’s multiple comparison test. The asterisk and pound represents statistically significant difference (*p < 0.05, **p < 0.01 compared to the control group; ^#^*p* < 0.05, ^##^*p* < 0.01 compared to the model group)

As shown in Fig. 3A, B and C, high-dose of PS-NPs (NPs-H) significantly increased the serum levels of CHOL and GLUC when compared to the model group indicating PS-NPs further disturbed glucose and lipid metabolism in mice with chronic colitis.

### PS-NPs treatment exacerbates liver oxidative stress in mice with DSS-induced chronic colitis

As shown in Fig. 3D, E, F and G, significantly elevated MDA level and significantly decreased SOD content was observed in the liver of mice from the model group compared to the control group, while the contents of GSH and T-AOC were not significantly different between the control group and model group. In mice with chronic colitis, treatment with PS-NPs (especially high doses of PS-NPs) further dramatically increased MDA level and significantly decreased the contents of SOD, GSH and T-AOC in the liver. However, 5-ASA treatment failed to significantly improve oxidative stress in the liver of mice with chronic colitis.

### PS-NPs treatment exacerbates intestinal colonic inflammation and immune response in mice with DSS-induced chronic colitis

**Fig. 4:**
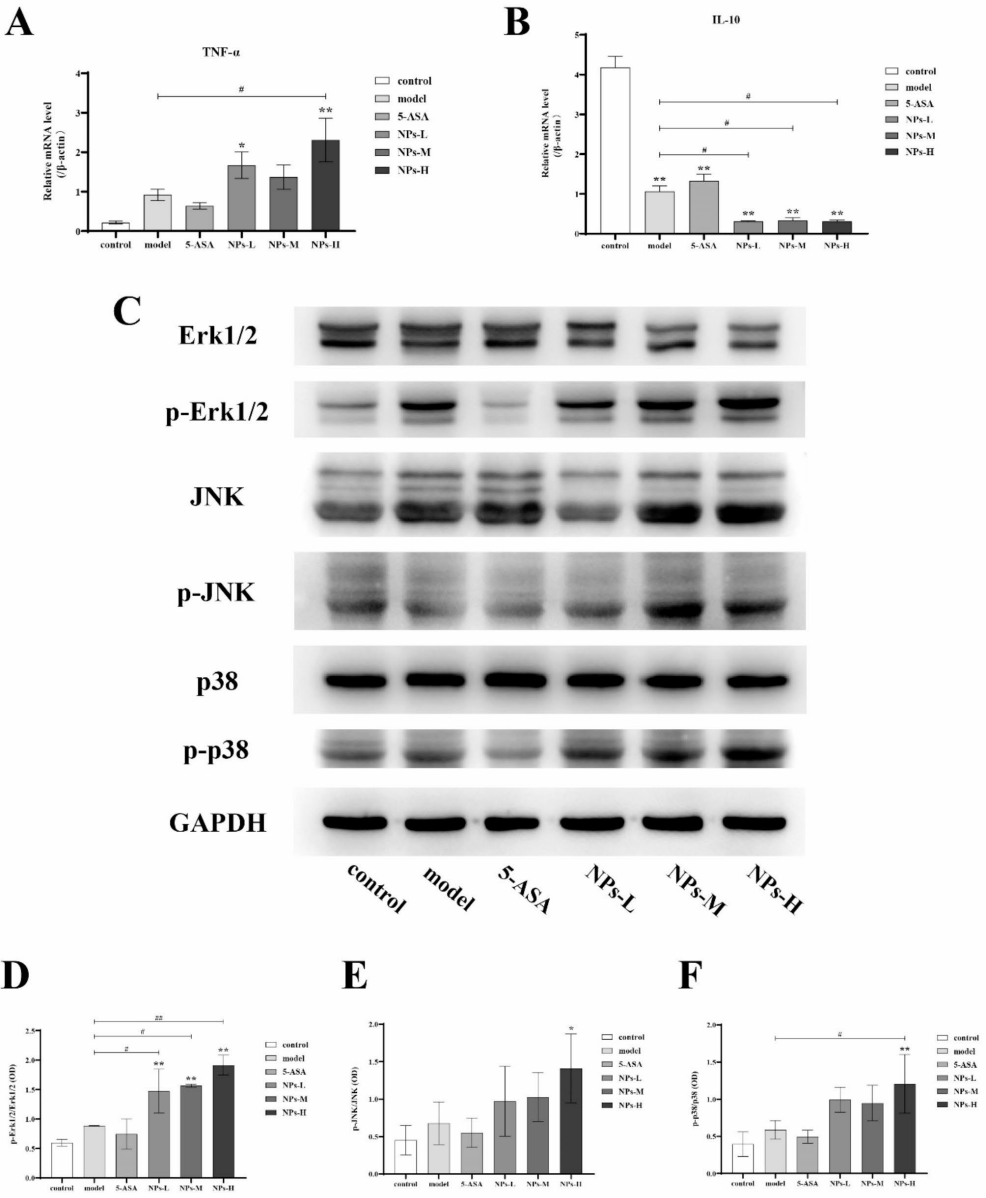
Expression levels of inflammatory factors and MAPK signaling pathway-related proteins in the colon. (**A**) TNF-α mRNA. (**B**) IL-10 mRNA. (Data are presented as mean ± SEM, n ≥ 6. One-way ANOVA and Multiple comparisons). (**C**) Expression of Erk1/2, p-Erk1/2, JNK, p-JNK, p38 and p-p38 proteins. (**D**) The relative densitometric analysis of p-Erk1/2 and Erk1/2. (**E**) The relative densitometric analysis of p-JNK and JNK. (**F**) The relative densitometric analysis of p-p38 and p38. Data are presented as mean ± SD, n = 3. One-way ANOVA and Tukey’s multiple comparison test. The asterisk and pound represents statistically significant difference (*p < 0.05, **p < 0.01 compared to the control group; ^#^*p* < 0.05, ^##^*p* < 0.01 compared to the model group)

The expression of mRNA of tumor necrosis factor (TNF)-α and interleukin (IL)-10 in colon tissue were measured by real-time fluorescence quantitative PCR using β-actin as an internal reference gene. As shown in Fig. 4A and B, compared to normal mice, the mRNA expression of TNF-α slightly increased while that of IL-10 significantly decreased in mice with chronic colitis. After treatment with various doses of PS-NPs, both rising TNF-α mRNA expression and declining IL-10 mRNA expression in mice with chronic colitis significantly aggravated, which was opposite to that of 5-ASA. These results indicated that PS-NPs exacerbated intestinal inflammation in mice with chronic colitis by promoting the expression of TNF-α mRNA and inhibiting the expression of IL-10 mRNA.

### PS-NPs treatment aggravates DSSinduced chronic colitis in mice by regulating the MAPK signaling pathway

MAPK signaling pathway is mainly composed of three protein kinases, namely C-Jun N-terminal kinase (Jnk), extracellular signal-regulated kinase (Erk1/2) and P38 [24]. MAPK pathway is closely related to cell proliferation [25], oxidative stress [26], inflammation [27] and other responses. So we analyzed if the MAPK signaling pathway is involved in PS-NPs-treated mice with chronic colitis. As shown in Fig. 4C, compared to normal mice, the protein expression ratio of p-Erk1/2 / Erk1/2, p-JNK/JNK and p-p38/P38 increased in the colon tissue of mice with chronic colitis (the model group). Opposite to that of 5-ASA, PS-NPs further increased the expressions of phosphorylated protein, indicating the exacerbating effect of PS-NPs on mice with chronic colitis associated with the regulation of the MAPK signaling pathway(Fig. 4D, E and F).

### PS-NPs treatment disturbes liver metabolism and lipidom in mice with DSS-induced chronic colitis

**Fig. 5:**
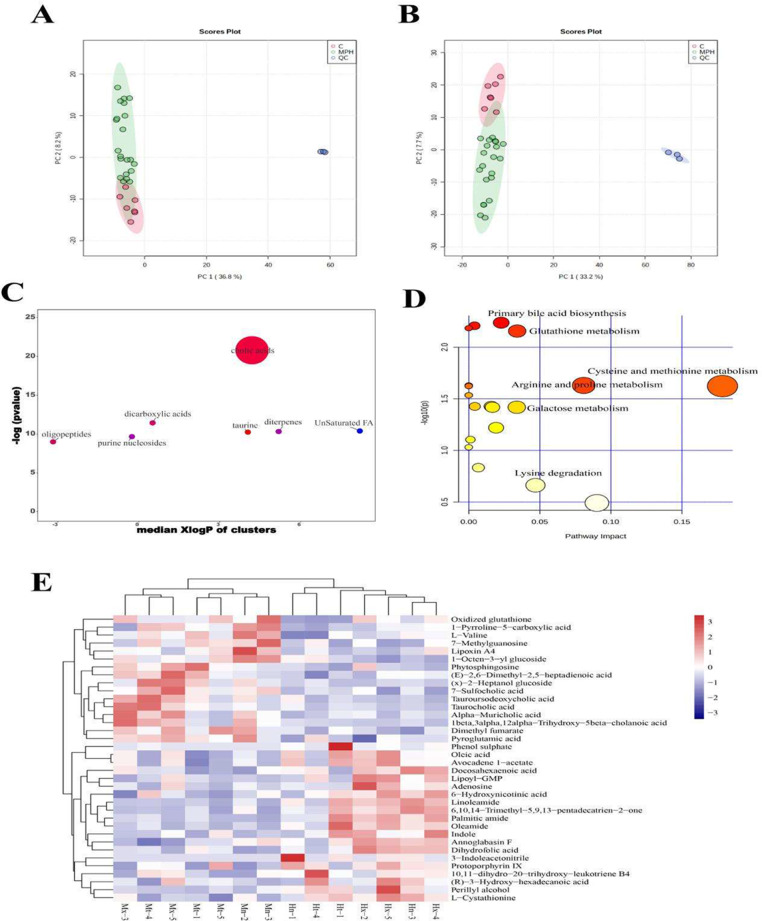
The liver metabolomics analysis in mice. **A** and **B**: PCA score plots of ESI +  and ESI −  modes of UPLC-Q-TOF, respectively (In the figure, C represents control group, M represents model group, P represents 5-ASA positive control group, and H represents NPs-H high dose group). **C**: The ChemRICH enrichment plots illustrated the markedly affected metabolites clusters in this study (*p *< 0.05). The plot y-axis represented the remarkably changed clusters. The total number of metabolites was represented by the sizes of the node. The colors of the cluster indicated the trend of increased or decreased metabolites (red = totally increased, blue = totally reduced, fuchsia = both but major increased, dark orchid = both but major decreased). **D**: Pathway analysis of 64 differential metabolites in the liver. **E**: Heat map of 36 differential metabolites between model and NPs-H groups

Metabolomic analysis of liver samples was performed using UPLC-Q-TOF/MS. After processing the data by Progenesis QI (Waters, America) and QI-met software, 6709 and 3866 ions were detected in positive and negative ion modes, respectively. With MetaboAnalyst 5.0 metabolomic data analysis platform, we imported the abundance values of all experimental samples and quality control (QC) samples for analysis. Figure 5A, B are PCA plots of positive and negative ions for the control group, the model group, the 5-ASA group, the NPs-H group and QC samples, respectively. As can be seen in the figure, the QC samples all converged tightly together, which indicated that the PCA model was reliable, suggesting that the assay system was well stabilized and the generated data results were credible. The abundance values of samples from the model group and the NPs-H group were imported using the MetaboAnalyst 5.0 metabolomic data analysis platform for screening analysis and difference investigation. The final 64 differential metabolites were screened using the Human Metabolome Database (HMDB) based on the criteria of VIP > 1, *p* < 0.05. The differential metabolites were mainly involved in bile acids, carboxylic acids, unsaturated fatty acids, diterpenoids, and taurine. ChemRICH enrichment plots depicted the significantly affected metabolite clusters, and the size of the nodes indicated the total number of metabolites. As can be observed in Fig. 5C, bile acids were the most significantly altered compounds in liver metabolism of mice from the model and the NPs-H group, with a metabolite cluster size of 6, while all other classes had a lower metabolite size of 3. Pathway analysis was further performed on the differential metabolites using the KEGG database. Figure 5D showed that the metabolic pathways of cysteine and methionine have the greatest effect on hepatic metabolism. Based on the ROC curve plotting, sensitivity and specificity analysis of the 64 differential metabolites were performed and 36 potential biomarkers were finally screened (Fig. 5E). According to all these analyses, we found that it is crucial to study the effects of NPs on hepatic lipid metabolism based on the chronic colitis model.

**Fig. 6:**
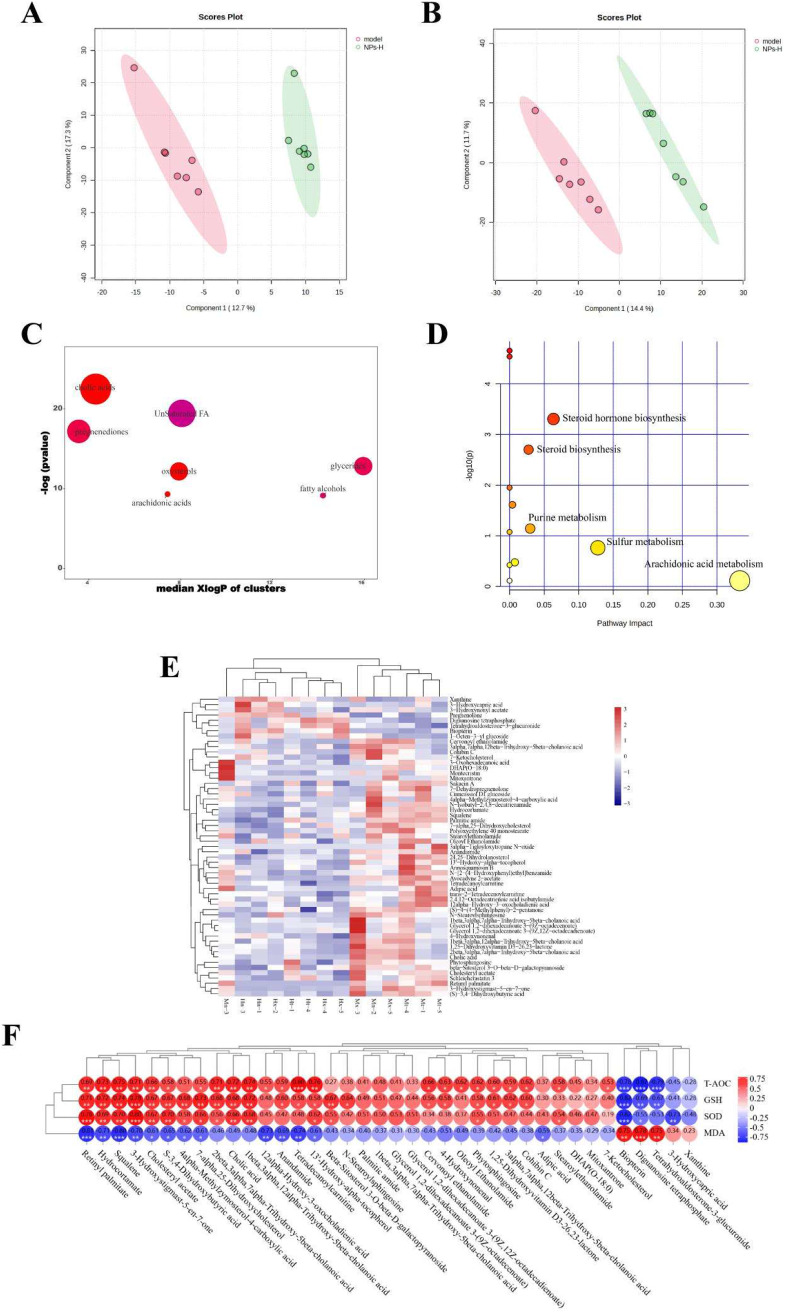
The liver lipidomic analysis in mice. **A** and **B**: PLS-DA score plots between the model group and the NPs-H group in the positive (a, R²=0.99449, Q²=0.67579) and negative (b, R²=0.99944, Q²=0.53059) modes, respectively. **C**: The ChemRICH enrichment plots illustrated the markedly affected lipids metabolites clusters in this study (*p* < 0.05). The plot y-axis represented the remarkably changed clusters. The total number of metabolites was represented by the sizes of node. The colors of the cluster indicated the trend of increased or decreased metabolites (red = totally increased, blue = totally reduced, fuchsia = both but major increased, dark orchid = both but major decreased). **D**: Pathway analysis of 88 differential lipids in the liver. **E**: Heat map of 57 differential lipids between the model group and the NPs-H group. **F**: Pearson correlation analysis between oxidative stress index and 38 differential lipids of the liver. Red = positively relevant, blue = negatively relevant. (*p < 0.05, **p < 0.01, ***p < 0.001)

The hepatic lipid metabolism of livers from mice of each group was further performed using UPLC-Q-TOF/MS. After processing the data by Progenesis QI and QI-met software, 5708 and 5458 ions were detected in positive and negative ion modes, respectively. The metabolite differences between samples from the model group and the NPs-H group were analyzed using partial least squares discriminant analysis (PLS-DA). Figure 6A, B showed the R² (model goodness of fit) and Q² (predictive power of the model) for the positive (a, R²=0.99449, Q²=0.67579) and negative (b, R²=0.99944, Q²=0.53059) models, indicating that the PLS-DA plots are reliable. We could see that the metabolites between the model group and the NPs-H group were significantly different by the scatter plot distribution of PLS-DA. 88 differential lipids, which were primarily involved in bile acids, unsaturated fatty acids, pregnenediones, triglycerides and oxidized cholesterol were screened based on the criteria of VIP > 1, *p* < 0.05. Figure 6C was the ChemRICH enrichment map of these 88 differential lipids, in which the most affected metabolite cluster was bile acid, followed by unsaturated fatty acids. The sulfur metabolic pathway was identified as a crucial route for lipid metabolism by the bubble pathway map of the lipid metabolism pathway that was analyzed using MetaboAnalyst 5.0 (Fig. 6D). Based on the analysis of 88 differential lipids using the ROC curve plotting, 57 potential biomarkers were finally identified and displayed in a heat map so that the clustering outcomes could be viewed (Fig. 6E). Based on the Pearson correlation analysis between the oxidative stress index and 38 differential lipids of the liver, we discovered that the key differential lipids were strongly linked to oxidative stress in the liver (Fig. 6F).

## Discussion

Inflammatory bowel disease (IBD) is a chronic gastrointestinal inflammatory illness characterized by innate immune system dysfunction mediated by toll-like receptors (TLRs) [28]. To further study the etiology and therapies of IBD, animal models are excellent tools because they allow for operations and therapies that are not possible in human studies [29]. The most often utilized models are acute and chronic colitis induced by DSS, and their etiology involves direct damage to the colon’s monolayer of epithelial cells, allowing intestinal contents to infiltrate underlying immune tissues and cause an inflammatory response [30]. The DSS model of chronic colitis and human IBD have similar immune pathogenesis of slow colonic healing followed by persistent immune activation mediated by both Th1 and Th2 cytokines. Notably, chronic colitis models can better mimic the real condition of human IBD than acute colitis models due to the persistent damage of intestinal epithelium [31]. In this study, to establish a chronic colitis model, healthy male C57BL/6J mice were cyclically fed a low dosage of DSS as previously reported. The chronic colitis in mice was characterized by significant decreases in colon length, and greater liver index, indicating that the liver is swollen, congested, and hypertrophy, which is consistent with the histopathology findings from the ongoing study. These findings revealed that the enteritis model was effective. The results demonstrated that PS-NPs exposure increased liver weight in DSS mice suggesting an enlargement of the liver. Further histological examination revealed marked intestine epithelial damage, turbidity swelling hepatocytes with enlarged fat vacuoles, and severe inflammatory infiltration of the colon and liver.

Cytokines are important mediators of inflammation, the mRNA level of TNF-α was elevated, while the mRNA level of anti-inflammatory cytokines IL-10 was decreased in the colonic tissue in DSS mice when compared to control mice. The findings are consistent with those of numerous previous studies that used varied concentrations and duration of DSS in different animal species, implying that they can be used as a credible index for these models [32–34]. Correspondingly, the exacerbation of inflammation in colon tissue by PS-NPs was confirmed by elevated TNF-α mRNA expression, decreased IL-10 mRNA expression, as well as results of H&E-staining research. Regarding the upstream mechanism, protein expression analysis based on western blot was investigated further. As aforementioned, MAPK signaling pathway is involved in the regulation of environmental factors on DSS-induced colitis in mice [35, 36]. PS-NPs exacerbate lipopolysaccharide-induced inflammation and necrosis in the mouse spleen via the ROS/MAPK signaling pathway [37]. Based on previous findings that small MPs (< 150 μm) may penetrate the gut epithelium [38], we hypothesized that 100 nm of PS-NPs studied here could exacerbate intestinal immune dysfunction and inflammation in mice with chronic colitis by penetrating the gut epithelium, increasing intestinal permeability, and inducing oxidative stress via the MAPK signaling pathway.

Liver is an important organ for regulating blood glucose level [39], which closely associated with oxidative stress and metabolic syndrome [40]. Currently, the majority of current research on the harmful effects of NPs on the liver is focused on marine fish. PS-NPs were discovered to be bioaccumulative in fish liver [41], to cause severe hepatic inflammation in zebrafish larvae [42], and to cause oxidative stress in the liver of juvenile L. crocea [43]. However, given to their pervasiveness, it is vital to investigate the effects of NPs on mammalian livers. We found that PS-NPs significantly increased the blood glucose (GLUC) content in the liver of the mice with chronic colitis, which in turn increased the level of cholesterol (CHOL). Liver tissues from mice with chronic colitis were further subjected to hepatic metabolomic analysis based on LC-MS. 16 metabolites were significantly upregulated and 20 were significantly downregulated after treatment with PS-NPs. PS-NPs had a significant upregulatory effect on bile acids, whose synthesis is a key metabolic pathway in cholesterol metabolism and plays an important role in hepatic lipid homeostasis [44]. Increased levels of bile acids in the liver were likewise found in patients with steatohepatitis [45]. Aside from bile acids, dicarboxylic acids, taurine and oligopeptides were significantly up-regulated, while unsaturated fatty acids were significantly down-regulated. Additionally, PS-NPs treatment drastically reduced the metabolite of tauroursodeoxycholic acid, an endoplasmic reticulum stress inhibitor that inhibits ERK phosphorylation [46] and significantly reduces the expression of apoptotic molecules such as caspase-3 and caspase-12 [47]. Similar result has been existed in previous study that chronic PVC MPs exposure causes hepatotoxicity and intestinal flora dysbiosis in mice [48].

In our invetstigation, PS-NPs exposure caused an increase in MDA level and decrease in GSH, SOD, T-AOC levels in liver of mice with chronic colitis. Through metabolic pathway analysis, the levels of T-AOC, GSH and SOD in the organism were found to had a negative correlation with 33 lipid differential metabolites and a positive correlation with 5, while the correlation between MDA level and hepatic lipid differential metabolites had opposite trends. PS-NPs significantly influenced the cysteine and methionine metabolic pathway that is a critical factor in GSH synthesis [49], resulting in reduced GSH content. The downregulation of oxidized GSH metabolites may be related to the decrease of GSH content in the liver, as the amount of GSH in the organism exists in both reduced and oxidized forms, oxidized GSH is produced by the oxidation of GSH [50]. The ChemRICH enrichment plots also showed that cholic acids, unsaturated fatty acids, pregnenediones, glycerides, oxysterols, arachidonic acids, and fatty alcohol metabolite clusters were significantly upregulated after PS-NPs treatment. In addition, many differential lipid metabolites that play an important role in the physiological processes of organisms such as squalene, anandamide, phytosphingosine and mitoxantrone were also identified in this study. Therefore, these findings suggested that PS-NPs exposure could worsen the secondary liver injury and contribute to the development of chronic liver disease in mice with chronic colitis. However, the complex relationship between oxidative stress, inflammation, metabolic disorders and PS-NPs still need to be conducted.

Collectively, exposure to PS-NPs with a diameter of 100 nm can worsen the health of mice with chronic colitis. PS-NPs exacerbated intestinal histopathology and inflammation in mice with chronic colitis by activating the MAPK signaling pathway. Furthermore, PS-NPs aggravated oxidative stress and intestinal permeability, which were tightly associated with hepatic lipid metabolism disturbance in the liver of mice with chronic colitis. Our study highlighted chronically ill populations’ susceptibility to environmental haza stress rds, which urgent more research and risk assessment studies. However, it has been shown that mice have different pulmonary toxicity responses to three different MPs [51], subsequent research should be based on real environmental situations, and some other types and shapes of MPs should be studied.

### Electronic supplementary material

Below is the link to the electronic supplementary material.

**Supplementary Material 1:** Western Blot picture

## Data Availability

Data is contained within the article.
