# Supplementary material for: Polystyrene nanobeads exacerbate chronic colitis in mice involving in oxidative stress and hepatic lipid metabolism
Source: Part Fibre Toxicol. 2023 Dec 18;20:49. doi: 10.1186/s12989-023-00560-8 (PMC10726634; doi:10.1186/s12989-023-00560-8)
Supplement: Supplementary file 1 — Supplementary Material 1: Western Blot picture [file 12989_2023_560_MOESM1_ESM.docx]

**
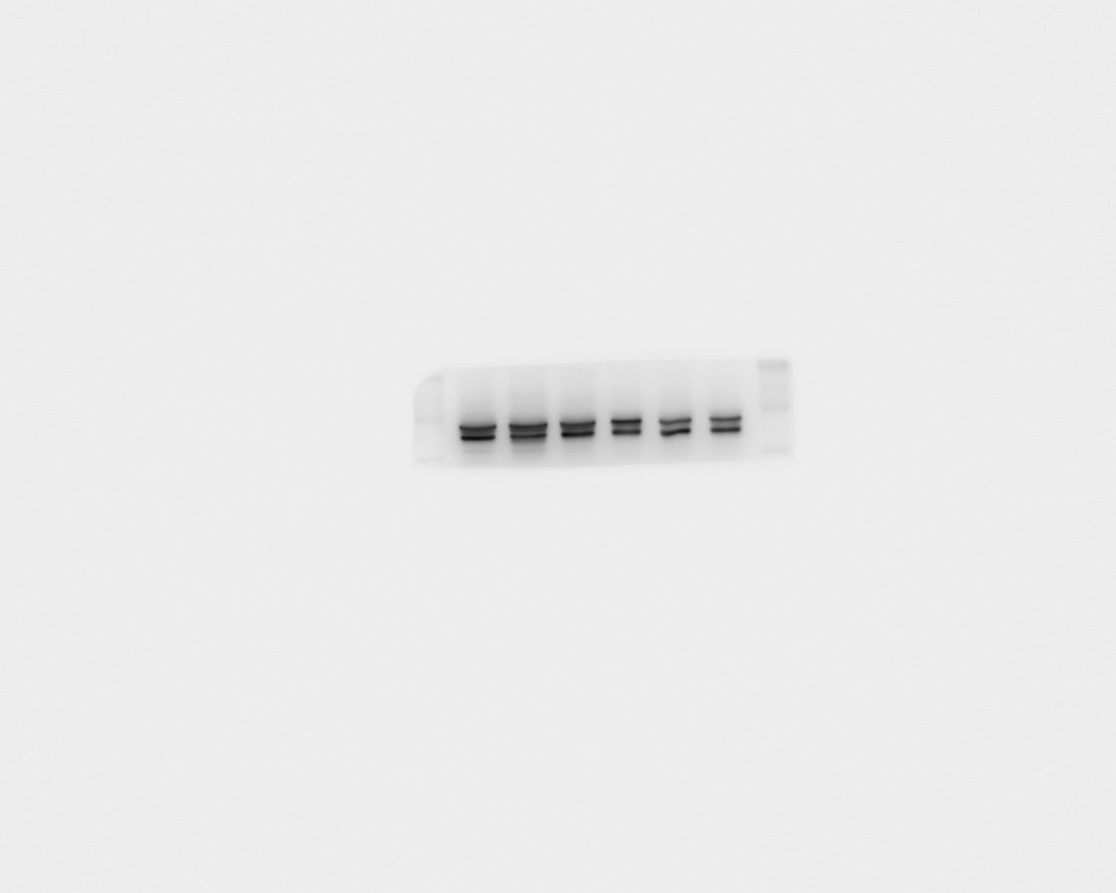
Western Blot picture**

**1. Protein: Erk1/2**

**Protein samples from left to right: control, model, 5-ASA, MPs-L, MPs-M, MPs-H**

**
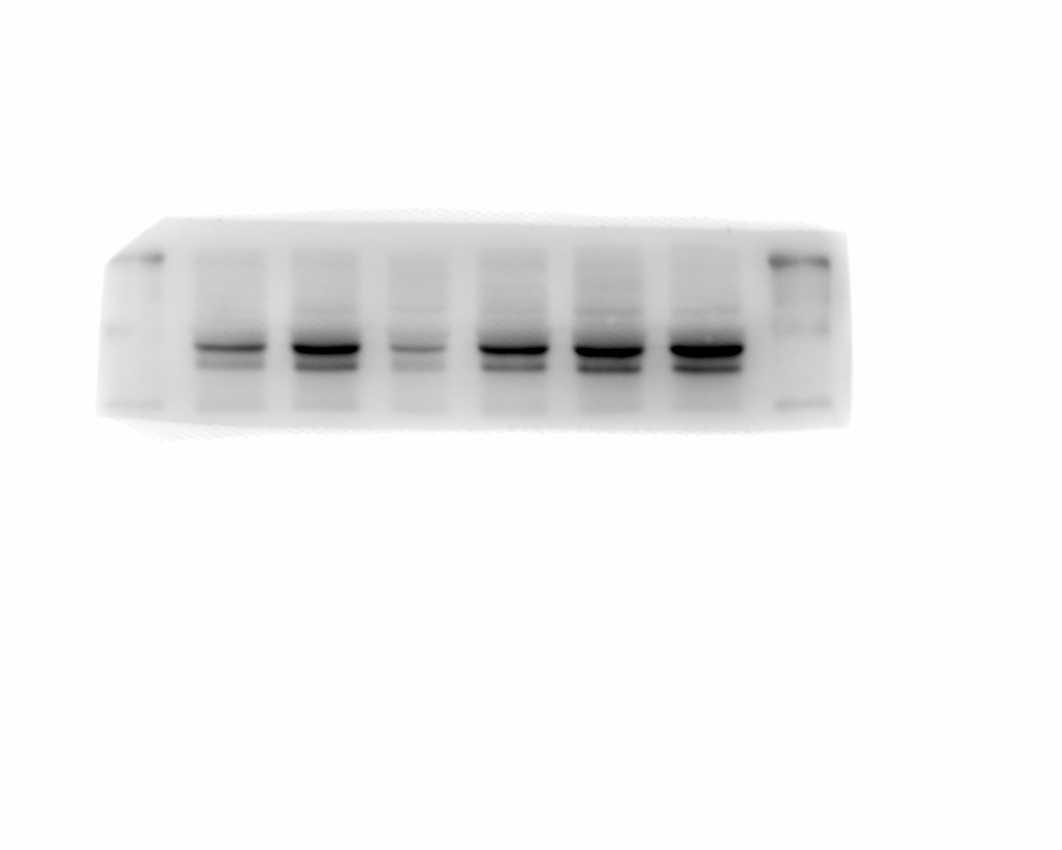
**

**2. Protein: p-Erk1/2**

**Protein samples from left to right: control, model, 5-ASA, MPs-L, MPs-M, MPs-H**

**
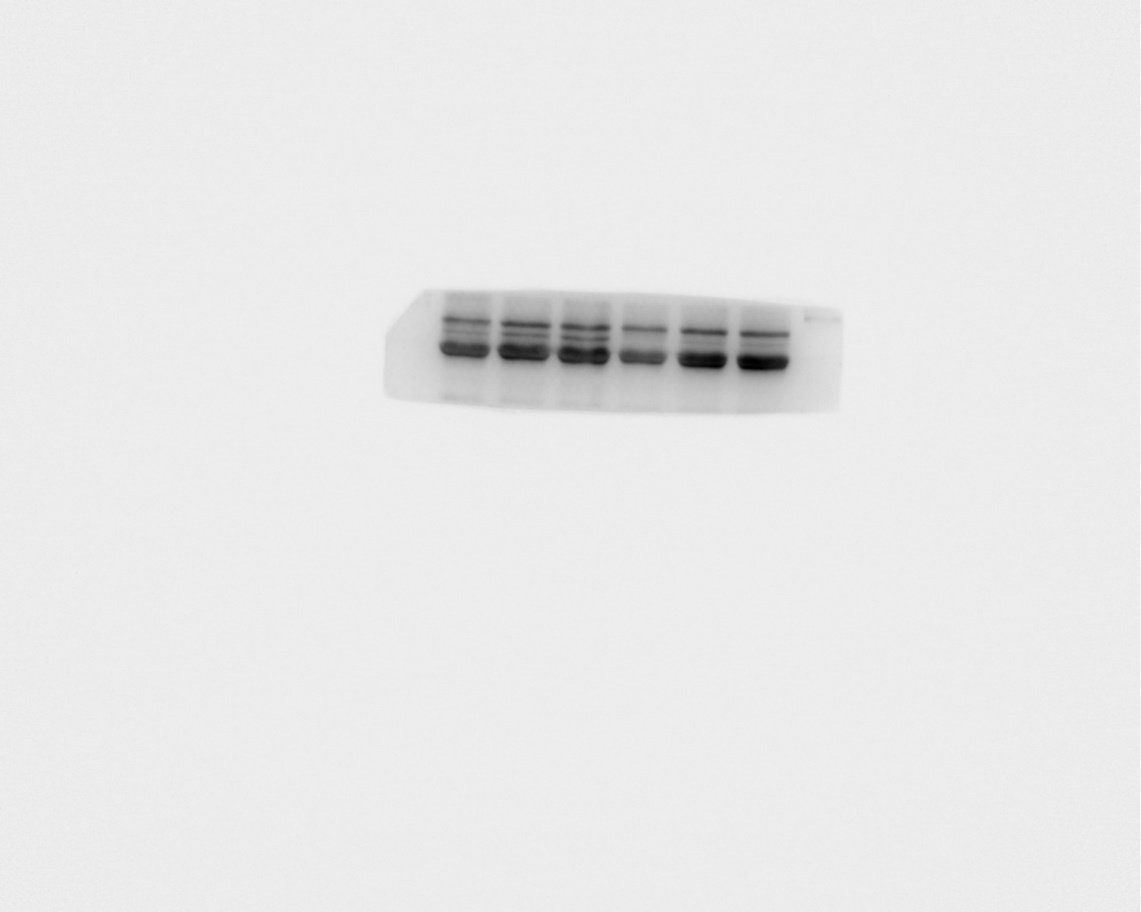
**

**3. Protein: JNK**

**Protein samples from left to right: control, model, 5-ASA, MPs-L, MPs-M, MPs-H**

**
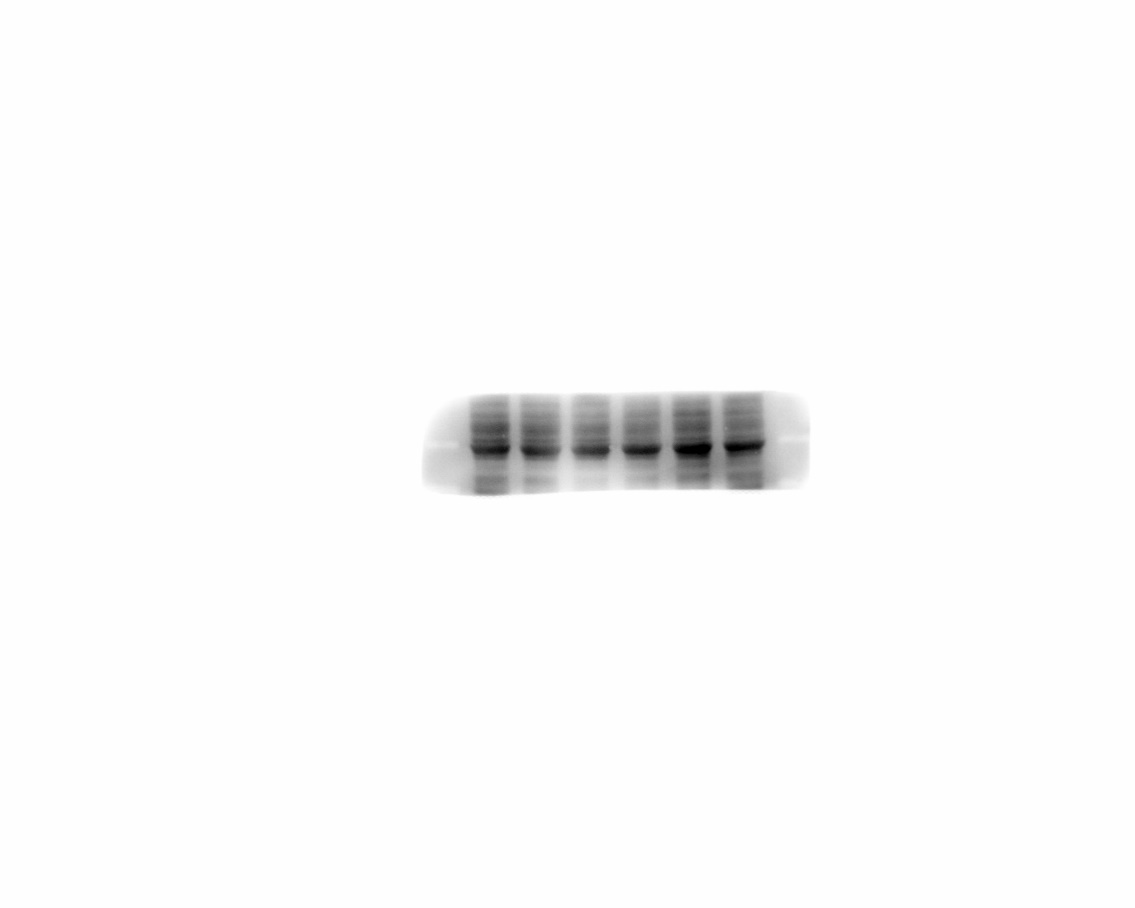
**

**4. Protein: p-JNK**

**Protein samples from left to right: control, model, 5-ASA, MPs-L, MPs-M, MPs-H**

**
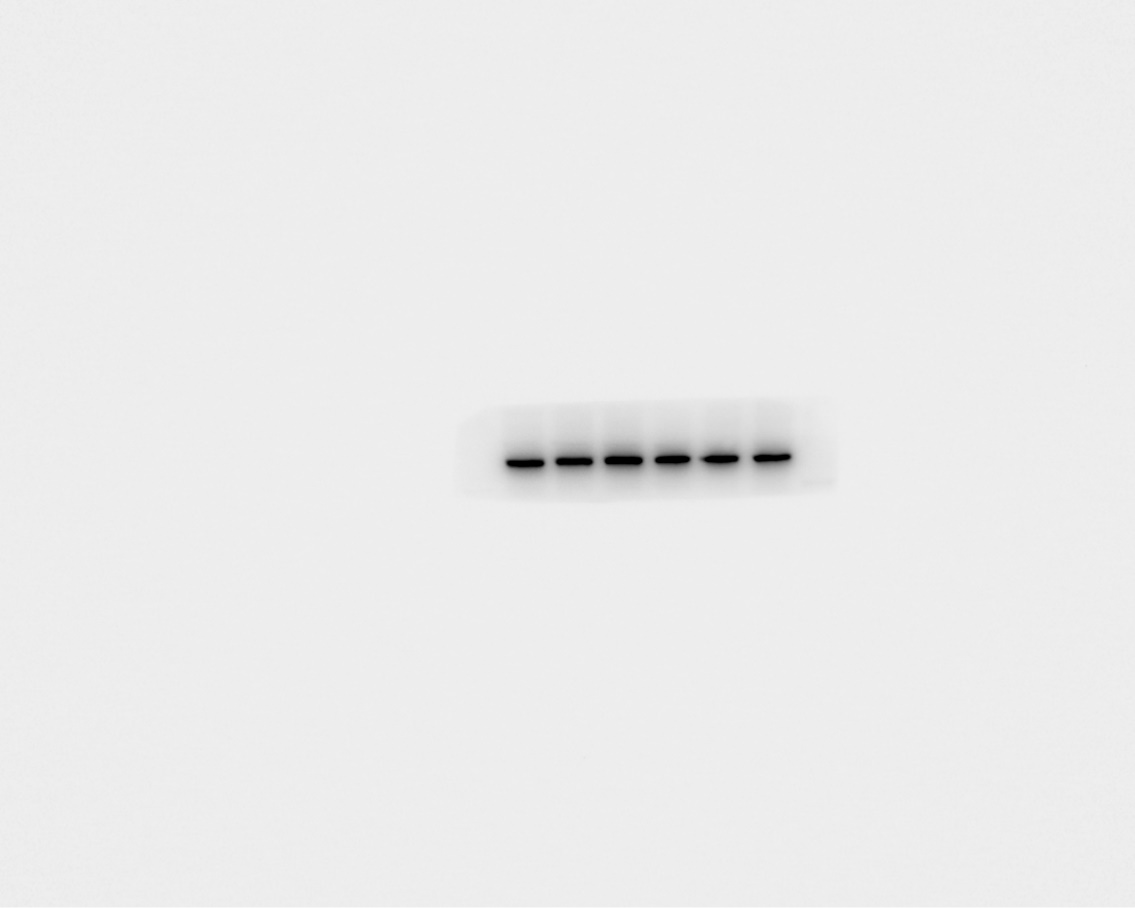
**

**5. Protein: p38**

**Protein samples from left to right: control, model, 5-ASA, MPs-L, MPs-M, MPs-H**

**
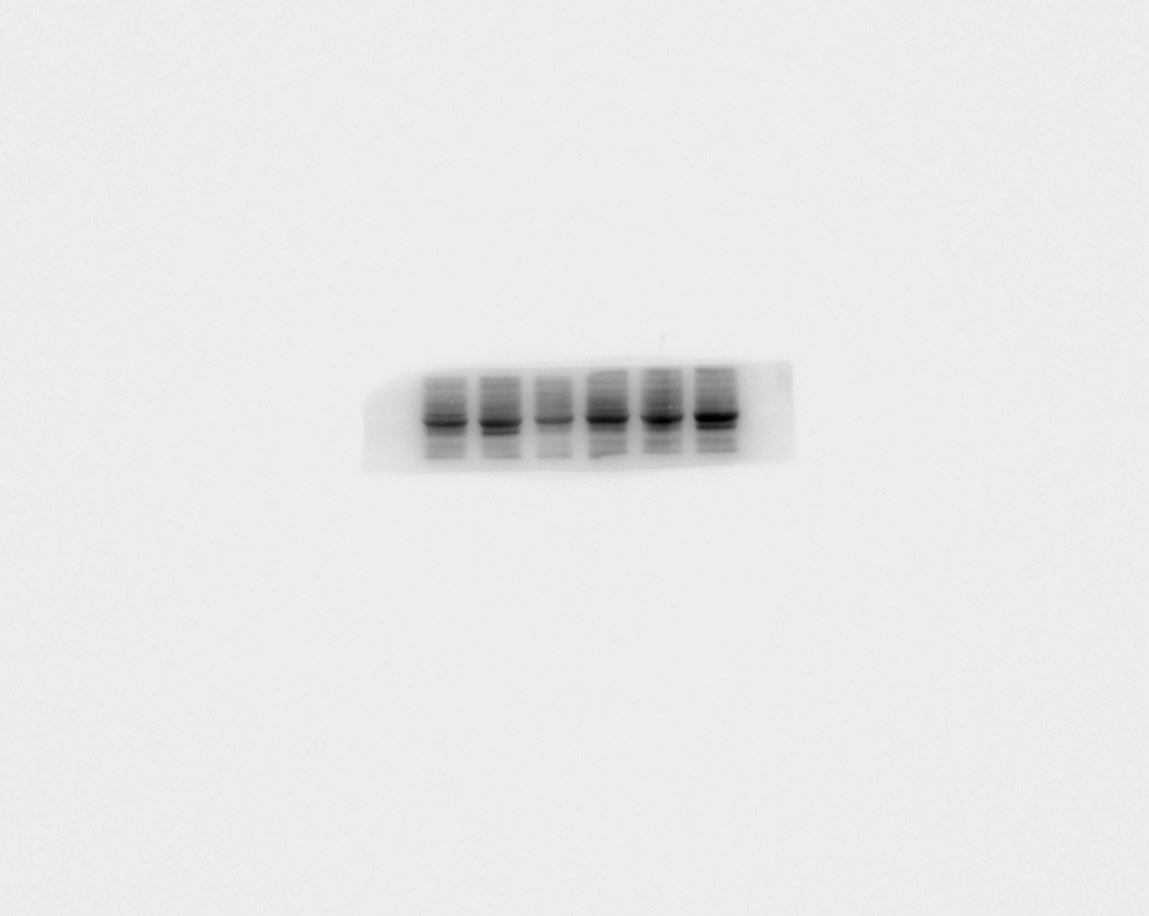
**

**6. Protein: p-p38**

**Protein samples from left to right: control, model, 5-ASA, MPs-L, MPs-M, MPs-H**

**
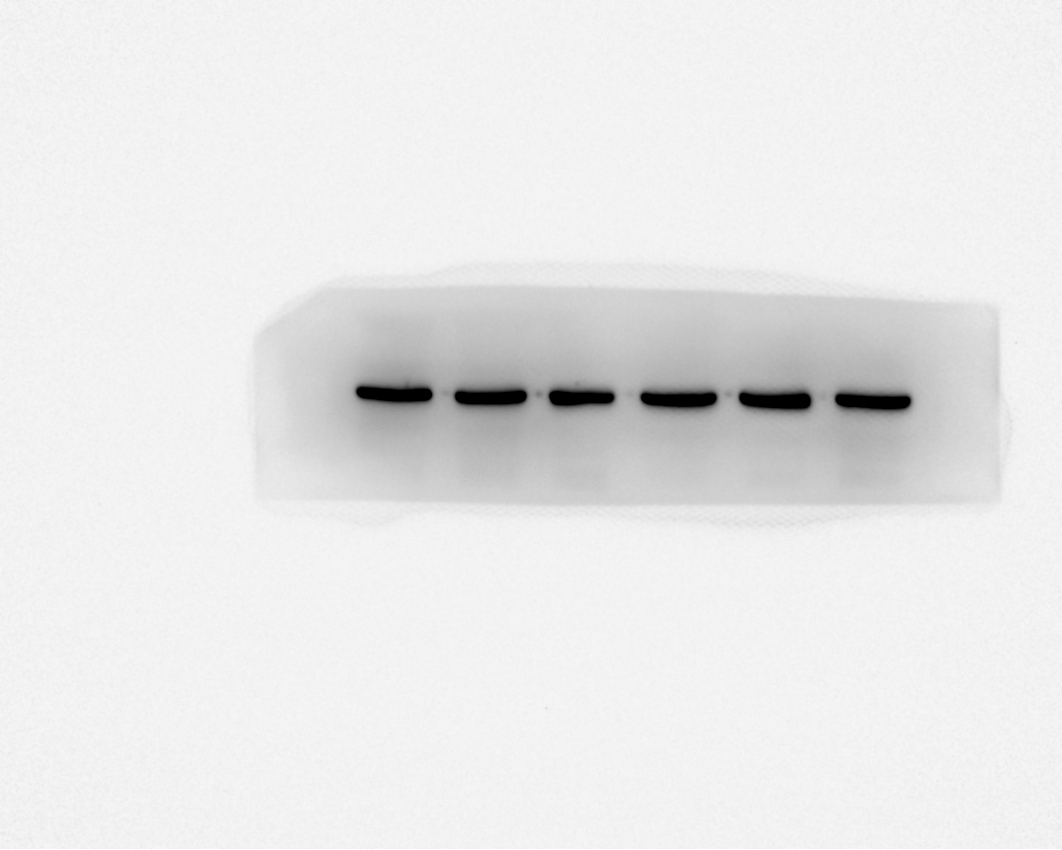
**

**7. Protein: GAPDH**

**Protein samples from left to right: control, model, 5-ASA, MPs-L, MPs-M, MPs-H**
